# Enrolling Incident Caregivers and Matched Controls From a Nationwide Epidemiological Study

**DOI:** 10.1093/geroni/igaa057.2272

**Published:** 2020-12-16

**Authors:** David Roth, William Haley, Orla Sheehan, J David Rhodes, Virginia Howard

**Affiliations:** 1 Johns Hopkins University, Baltimore, Maryland, United States; 2 University of South Florida, Tampa, Florida, United States; 3 Johns Hopkins University School of Medicine, Baltimore, Maryland, United States; 4 University of Alabama at Birmingham, Birmingham, Alabama, United States

## Abstract

Participants in the national Reasons for Geographic and Racial Differences in Stroke (REGARDS) study were asked about family caregiving responsibilities at enrollment (2003-2007). Among the 88% of participants who were not caregivers at enrollment, 1,229 reported becoming caregivers before a follow-up interview 12 years later. The Caregiving Transitions Study screened these participants and enrolled 251 as incident caregivers. All reported 5 or more hours of care per week, provided assistance with at least one ADL or IADL, and were caregivers for at least 3 months before a 2nd blood sample was obtained in the REGARDS study. A total of 251 noncaregiving control participants who reported no caregiving responsibilities over this 12-year period were also enrolled. Each control was matched to a caregiver on age (+ 5 years), sex, race, other demographics, and baseline (pre-caregiving) health variables. Descriptive analyses confirm the unique comparability of the samples compared to previous caregiving studies.

